# Determination of the Interaction and Pharmacological Modulation of MCHR1 Signaling by the C-Terminus of MRAP2 Protein

**DOI:** 10.3389/fendo.2022.848728

**Published:** 2022-03-04

**Authors:** Meng Wang, Yue Zhai, Xiaowei Lei, Jing Xu, Bopei Jiang, Zhe Kuang, Cong Zhang, Shangyun Liu, Shan Bian, Xiao-Mei Yang, Tao Zan, Li-Na Jin, Qingfeng Li, Chao Zhang

**Affiliations:** ^1^ Department of Plastic and Reconstructive Surgery, Shanghai Institute of Precision Medicine, Shanghai Ninth People’s Hospital, Shanghai Jiao Tong University School of Medicine, Shanghai, China; ^2^ Fundamental Research Center, Shanghai Yangzhi Rehabilitation Hospital (Shanghai Sunshine Rehabilitation Center), School of Life Sciences and Technology, Tongji University, Shanghai, China; ^3^ Department of Hematology, Changzheng Hospital, Naval Medical University, Shanghai, China; ^4^ Institute for Regenerative Medicine, Shanghai East Hospital, School of Life Sciences and Technology, Tongji University, Shanghai, China

**Keywords:** MRAP2, GPCR, melanin-concentrating hormone, pharmacological regulation, MCHR1

## Abstract

Melanin concentrating hormone (MCH), an orexigenic neuropeptide, is primarily secreted by the hypothalamus and acts on its receptor, the melanin-concentrating hormone receptor 1 (MCHR1), to regulate appetite and energy homeostasis. The Melanocortin Receptor Accessory Protein 2 (MRAP2), a small single transmembrane protein broadly expressed in multiple tissues, has been defined as a vital endocrine modulator of five melanocortin receptors (MC1R–MC5R) and several other GPCRs in the regulation of central neuronal activities and peripheral energy balance. Here, we demonstrated the interaction between MRAP2 and MCHR1 by immunoprecipitation and bimolecular fluorescent assay and found that MRAP2 could inhibit MCHR1 signaling *in vitro*. A series of functional truncations of different regions further identified that the C-terminal domains of MRAP2 protein were required for the pharmacological modulation of intracellular Ca^2+^ coupled cascades and membrane transport. These findings elucidated the broad regulatory profile of MRAP2 protein in the central nervous system and may provide implications for the modulation of central MCHR1 function *in vivo*.

## Highlights

- MRAP2 interacts with MCHR1 and suppresses MCHR1 signaling *in vitro*.
- The C-terminal domain of MRAP2 is required for the intracellular Gq coupled Ca^2+^ cascades and membrane transport of MCHR1.
- These findings provide implications for the functional modulation of the central MCHR1–MRAP2 complex *in vivo*.

## Introduction

Hypothalamus functions as one of the most important neuronal cores for the integration of a variety of physiological signals from the periphery to modulate secretion of multiple peptidic pituitary hormones involved in maintaining energy homeostasis and proper feeding behavior. Melanin concentrating hormone (MCH), a cyclic peptide primarily expressed in hypothalamus, participates in the regulation of energy balance (1) through two G protein-coupled receptors, MCHR1 and MCHR2 (2). MCH binds to MCHR1 (also called SLC-1 or GPR24) with high affinity and MCHR1 has been shown to couple to three Gα proteins: Gi, Go, and Gq. In the hippocampus and frontal cortex, MCHR1 preferably signals through the Gq-coupled pathway to increase intracellular Ca^2+^ level (3). Deletion of MCHR1 creates a phenotype of weight loss and resistance to diet-induced obesity characterized by hyperphagia, hyperactivity, and hypermetabolism (4), and indicates a central role of MCHR1 in stimulating food intake and increasing body weight in rodents (5).

Melanocortin receptor accessory proteins (MRAPs) include two members, the melanocortin receptor accessory protein 1 (MRAP1) and melanocortin receptor accessory protein 2 (MRAP2). MRAP2 is highly expressed in the hypothalamus, especially the paraventricular nucleus (PVN), and has been linked to mammalian obesity syndromes (6, 7). It has been shown that MRAP2 potentiates melanocortin-4 receptor (MC4R) signaling in several species (6, 8, 9) and plays a key role in GPCR signaling to regulate the dynamic neuronal appetite and food intake. Both MRAP2- and MC4R-deficient backgrounds develop severe obesity syndrome in human and murine models (6, 8, 10, 11). Notably, MC4R KO mice exhibit a hyperphagia phenotype that is absent in MRAP2 knockout ones. Given that MRAP2 also regulates the activity of several other metabolic-related GPCRs besides MC4R, such as PKRs (12), OX1R (13), and GHSR1α (14), and the regulatory regions for these GPCRs are specific (13, 15), we speculate that the absence of hyperphagia in MRAP2 knockout mice could arise from the coordinated modulation of the MRAP2 protein on multiple endogenous metabolic-related GPCR signaling pathways.

In this study, we investigated the regulatory profile of the MRAP2 protein on the trafficking and signaling of MCHR1. MRAP2 interacted with MCHR1 *in vitro* and the pharmacological regulation of MCHR1 signaling required distinct functional regions of MRAP2 protein. Collectively, we observed that the C-terminus of MRAP2 was mainly responsible for the inhibition of MCHR1 signaling and cell surface translocation. Our data not only identified MCHR1 as a novel metabolic-associated GPCR target of the MRAP2 protein, but also elucidated the complex endocrine network of GPCR signaling, which may explain the composite metabolic phenotypes of MRAP2 deficient murine models.

## Methods and Materials

### *In Silico* Analysis of Bulk and Single Transcriptome of MCHR1, MRAP1, and MRAP2 Expression

Human bulk RNA-seq datasets were downloaded from the Genotype-Tissue Expression (GTEx) project (https://www.gtexportal.org/) and mouse RNA-seq datasets were downloaded from all RNA-seq and ChIP-seq sample and signature search (ARCHS4) database (https://amp.pharm.mssm.edu/archs4/). Datasets of mouse cerebral and hypothalamic single-cell RNA-seq including GSE74672, GSE87544, GSE130597, and GSE125065 were downloaded from Gene Expression Omnibus (GEO) datebase.

For bulk RNA-seq analysis, we employed FPKM (Fragments per Kilobase Million) as the normalized value. Next, we analyzed the samples of whole brain and hypothalamus of humans and mice, respectively. We calculated Pearson’s correlation coefficient between MCHR1 and MRAP1 or MRAP2 expression value across all samples. For single-cell RNA-seq analysis, all datasets were filtered and low-quality cells with unique feature counts less than 200 were excluded. The co-expression of cell numbers of MRAP2 and MCHR1 positive neuronal populations was calculated. Next, Pearson’s correlation coefficient was calculated between the expression value of MRAP2 and MCHR1 across all cells.

To calculate the changes in MRAP2 and MCHR1 expression under high-fat diet condition, we counted the proportion of cells expressing these transcripts in the high-fat diet and normal chow condition, respectively. We also tested the significance of the differences using Fisher’s exact test. In order to explore the pathways in which these genes are involved, we performed GO pathway enrichment analysis using clusterProfiler R package.

### Reagents, Plasmids, and Antibodies

MCHR1, MRAP1, and MRAP2 were amplified from wild-type C57/BL6 mice cDNA library. All PCR products were ligated into pcDNA3.1(+) vector and the constructs were verified by Sanger sequencing. Melanin concentrating hormone (MCH) was purchased from BACHEM. SNAP-94847 (MCHR1 antagonist) was purchased from MCE (MedChemExpress). In the following assays, we purchased anti-mouse HA monoclonal antibody (Sigma-Aldrich, MO, USA), anti-mouse Flag monoclonal antibody (ABclonal Biotech Co., Ltd, Wuhan, China), anti-mouse IgG antibody (ABclonal Biotech Co., Ltd, Wuhan, China), and HRP-conjugated antibodies against mouse antigens (Abclonal Biotech Co., Ltd, Wuhan, China).

### Cell Culture and Transfection

HEK293T cells were cultured in DMEM medium (high glucose) supplied with 10% FBS and 1% penicillin–streptomycin (P/S). Cells were incubated in a 37°C incubator consisting of 5% CO_2_. Transfection was conducted using P-PEI reagent according to the manufacturer’s protocols. The total amount of transfected plasmids was kept identical in each group by adding empty pcDNA3.1 vector. The concentration of cells seeded for each single assay was 10^5^/ml.

### Tissue Expression Analysis

RT-PCR was performed as previously described (16). Briefly, cDNA was synthesized by extracting RNA from 14 mouse tissues (heart, liver, spleen, lung, stomach, pancreas, fat, kidney, brain, cerebellum, eye, thorax, spinal cord, and genital gland). All PCR products were separated on 1.5% agarose gel and β-actin was utilized as internal control. Primers used in this study were all synthesized from GENEWIZ (Shanghai, China) and the primer sequences of mMCHR1, mMRAP1, mMRAP2, and mβ-actin are listed as follows. mMCHR1_fw: ATCACTGCTGCGTACGTGAA; mMCHR1_rev: TCACCCTCTTTGTCCGAAGC; mMRAP1_fw: CTGAAAGCCAACAAGCATTCCA; mMRAP1_rev: CCGACCAGGACATGTAGAGC; mβ-actin _fw: GCCTTCCTTCTTGGGTATGGA; mβ-actin_rev: ACGGATGTCAACGTCACACT.

### Western Blot and Co-Immunoprecipitation

Proteins were extracted 24–36 h upon transfection and then incubated with mouse anti-HA or mouse anti-Flag antibody at 1:5,000 dilution overnight at 4°C. The next day, protein A+G beads (Beyotime, Shanghai, China) were added and rotated at 4°C for 4 h. Finally, beads were resuspended in protein loading buffer after three washes and boiled for 15 min. Samples were loaded on 12% SDS-PAGE gels and mouse anti-FLAG antibody was used for detecting MRAP1/MRAP2 in MCHR1 co-immunoprecipitation experiments. Images were captured by ImageQuant 4000.

### Bimolecular Fluorescence Complementation and Co-Immunofluorescence Assay

HEK293T cells were seeded on poly-L-lysine pretreated coverslips of 12-well plates and transfected with MCHR1-F1 and MRAP1-Flag-F2 or MRAP2-Flag-F2. Each well was transfected with 1 μg of plasmids in total. The next day, cells were fixed with 4% PFA Fix Solution for 20 min. Cells were incubated with anti-FLAG antibody (Cell Signaling) at 1:5,000 for 2 h at room temperature for the detection of MRAP1 and MRAP2 proteins. Then, samples were washed 3 times and incubated with 1:5,000 secondary antibody Alexa Fluor594 (Abcam) for 2 h at room temperature. To detect membrane translocation of MCHR1 in the presence of MRAP2, we transfected GFP-MCHR1 and MRAP2 or RAMP3 at a ratio of 1: 9 without antibody treatment. In order to observe co-fluorescence of MCHR1 and MRAP2 chimeras, 3HA-MCHR1 and each 2xFLAG MRAP2 chimera were transiently transfected. Cells were incubated with both anti-HA and anti-FLAG antibody (Cell Signaling) at 1:5,000 for 2 h at room temperature. Samples were then washed 3 times and incubated with both 1:5,000 secondary antibody Alexa Fluor488 (Abcam) and Alexa Fluor594 (Abcam) for 2 h at room temperature.

Coverslips were fixed with nail polish on the glass slide containing ProLong (R) Gold Antifade with DAPI Molecular Probes (Cell Signaling). Imaging was collected using a 63× oil objective with laser-scanning Zeiss confocal microscopy (LSM880).

### Enzyme-Linked Immunosorbent Assay

HEK293T cells were seeded in 12-well plates and transfected with MCHR1 and MRAP2 (1:0 to 1:9 ratio receptor to MRAP2). Each well was transfected with 1 μg of plasmids in total. Upon 24–36 h transfection, ELISA was performed as previously described (17). Cells were fixed 20 min with 4% PFA after 3 washes and then blocked with 5% milk in PBS for 30 min at room temperature. Next, cells were incubated with mouse anti-HA monoclonal antibodies (1:2,000) for 2 h, following HRP-conjugated secondary antibodies (1:2,000) incubation for 2 h at room temperature. After incubating with TMB solution for 15 min, the reaction was stopped with 5% sulfuric acid. The absorbance was measured at 450 nm on a Spectramax M5 multimode plate reader.

### Ca2+ Luminescent Assay

HEK293T cells were cultured in 24-well plates and different MRAP2 mutants were co-transfected along with MCHR1, NFAT (firefly luciferase), and pRL-TK (Renilla luciferase) reporter vectors *via* P-PEI according to the manufacturer’s instructions. A total of 1 μg of plasmid was transfected in each group of three wells. After 24–36 h transfection, medium was removed and different concentrations of MCH (from 10^-6^ to 10^-11^ M) in DMEM supplemented with 0.1% BSA were added and incubated for 9 h at 37°C. For competitive inhibition assay, a concentration of SNAP-94847 (MCHR1 antagonist) ranging from 10^-6^ to 10^-11^ M was added in HEK293T cells supplemented with 2 ×10^-9^M (EC80) MCH.

Dual-luciferase reporter assays were conducted using Dual-Glo kits (Promega, WI, USA) according to the manufacturer’s instructions. Luciferase activities were measured by a Spectramax M5 multimode plate reader. Firefly luciferase values were normalized to Renilla luciferase values for relative quantification.

### Sequence Homology Comparison

DNAMAN software was utilized to compare the protein sequence similarity of human and mouse MRAP2. Distinct colors highlighted the similarity score of various amino acids, in which red represented 100% consistency between sequences; blue indicated that the amino acids at this position were not conserved with a similarity score between 0 and 33%.

### Statistical Analysis

All experiments in this study were repeated at least three times. Data were analyzed by the GraphPad Prism6 software. Pharmacological curves were carried out by the log(agonist) *vs*. response equation (Y = Bottom+(Top-Bottom)/(1 + 10^((LogEC50–X))) (X: log(agonist); Y: response values) method. One-way ANOVA with Tukey post-test was applied to measure significance between groups, and results were shown as mean ± SEM. The tests were performed with a nominal significant level of *p < 0.05, **p < 0.01, ***p < 0.001, and ****p < 0.0001.

## Results

### Expressional Correlation of MCHR1 and MRAP2 Transcripts

**Figure 1:**
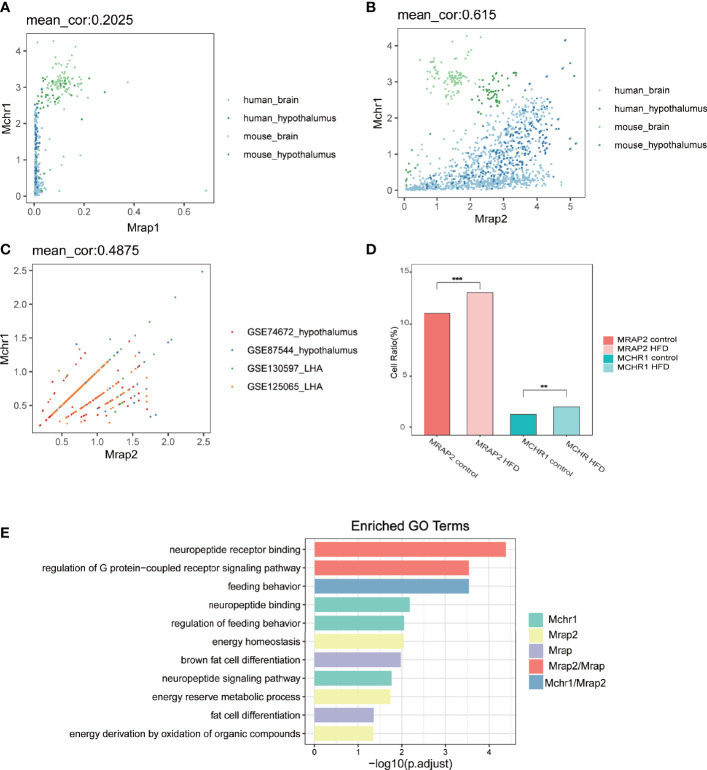
Co-expression and functional analysis of MCHR1 and MRAP proteins in human and mouse RNA-seq datasets. **(A, B)** Co-expression correlation analysis of MRAP1 **(A)** or MRAP2 **(B)** with MCHR1 from the bulk RNA-seq data of human or mouse brain and hypothalamus. **(C)** Correlation coefficient between MRAP2 and MCHR1 from four single-cell RNA-seq datasets of mouse hypothalamus. **(D)** Changes in cell proportion of MCHR1 and MRAP2 in different metabolic states. One-way ANOVA with post-hoc Tukey test. **p < 0.01, ***p < 0.001. **(E)** GO enrichment pathway analysis of MCHR1 and MRAP proteins.

First we developed a custom script to interrogate the co-expression correlation of MCHR1 and MRAP1 or MRAP2 in 1,698 human or mouse bulk RNA-seq samples of central nervous system (namely, 1,000 human whole brain samples, 435 mouse whole brain samples, 202 human hypothalamic samples, and 61 mouse hypothalamic samples) from the Genotype-Tissue Expression (GTEx) project databases and all RNA-seq and ChIP-seq sample and signature search (ARCHS4) database. As shown in **Figures 1A, B**, a few cells co-expressed MCHR1 and MRAP1 in bulk RNA-seq datasets with a low correlation (mean_cor = 0.2) between MCHR1 and MRAP1. While the number of co-expressing MCHR1 and MRAP2 is comparatively larger with a high correlation score (mean_cor = 0.6) between MCHR1 and MRAP2. The correlation between the two seems to be higher in the hypothalamus than other brain regions (**Figure 1B**). In order to better explore the physiological effect of MCHR1 on the phenotype of MRAP2 null mice and the actual co-expression of two transcripts within the same cell, we next analyzed the co-expression correlation of MCHR1 and MRAP2 in 28,320 cells of 20 mice from 4 published single-cell RNA-seq datasets of the Gene Expression Omnibus (GEO) database. We found that MCHR1 and MRAP2 co-expressed in most of neuronal cells in the central nervous system (**Figure 1C**). The average correlation between MCHR1 and MRAP2 in the 4 datasets was relatively large (mean_cor = 0.5).

MCHR1 and MRAP2 both played a critical role in the mammalian energy homeostasis. Therefore, we analyzed the cell number ratio change of MCHR1 and MRAP2 under different metabolic conditions. The results showed that MCHR1 and MRAP2 exhibited consistent cell ratio shift in the High-Fat Diet induced group compared to controls, which were both significantly upregulated (**Figure 1D**). Furthermore, we found that MRAP1, MRAP2, and MCHR1 were enriched in several metabolism associated pathways. Among them, MRAP1 and MRAP2 were both related to the binding of neuropeptides and GPCR signaling pathways. More importantly, GO (Gene Ontology) functional analysis found that both MCHR1 and MRAP2 contributed to the regulation of feeding behavior (**Figure 1E**). These results indicated that MCHR1 and MRAP2 could exhibit a synergistic effect on regulating energy metabolism in the central nervous system.

### Tissue Distribution and Interaction of MCHR1 With MRAP1 or MRAP2

**Figure 2:**
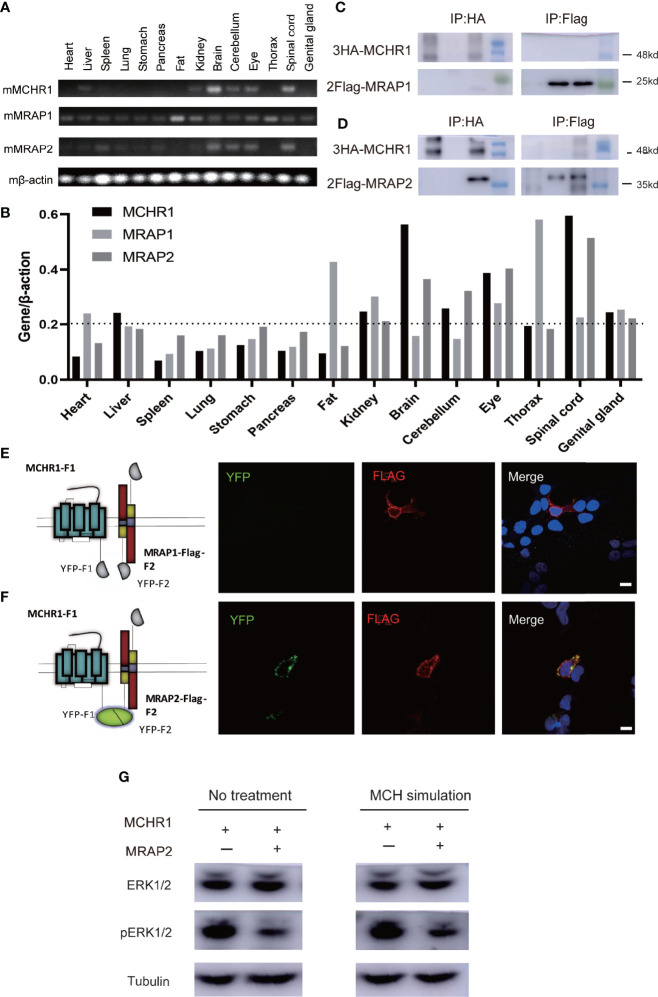
Protein interaction of MCHR1 with MRAP2 but not MRAP1. **(A)** Tissue distribution of MCHR1, MRAP1, and MRAP2 tested by RT-PCR. β-actin was used as an internal control. **(B)** Expression abundance analysis of MCHR1, MRAP1, and MRAP2 in 14 mouse tissues. **(C, D)** Co-IP analysis of the interaction between 3HA-MCHR1 and 2Flag-MRAP1 **(C)** or 2Flag-MRAP2 **(D)**. The numbers on the right indicate molecular weight of marker band on the right (kd). **(E, F)** MCHR1-F1 co-localizes with MRAP1-Flag-F2 or MRAP2-Flag-F2 in live cells. YFP fluorescence is exhibited in green (left panel). MRAP1 or MRAP2 in the same cells detected with anti-Flag antibody and secondary anti-mouse Alexa594 is shown in red (middle panel). DAPI were applied to stain cell nuclei and shown in blue in merge figures (right panel) (scale bars, 10 μm). **(G)** Western blot for ERK1/2 and pERK1/2, and Tubulin is used as reference. The sample order: MCHR1, MCHR1+MRAP2, MCHR1 with MCH simulated, MCHR1+MRAP2 with MCH simulated (from left to right).

To characterize mRNA expression profiles of MCHR1, MRAP1, and MRAP2 *in vivo*, RT-PCR analysis was extended to 14 tissues from adult mice (**Figures 2A, B**). The expression of MCHR1, MRAP1, and MRAP2 showed consistency in several tissues, such as brain, cerebellum, eye, and spinal cord. These results suggested that the MCHR1, MRAP1, and MRAP2 might function as a complex unit *in vivo*.

These findings provided actual evidence for the co-expression of these proteins *in vivo*. In order to further determine whether MRAP2 could interact with MCHR1 protein, we performed co-immunoprecipitation (Co-IP) assays in HEK293T cells transfected with 3HA-MCHR1 and 2Flag-MRAP1 or 2Flag-MRAP2. As shown in **Figures 2C, D**, MCHR1 barely co-purified with MRAP1 but strongly co-purified with MRAP2.

Next, to further assess the ability of MCHR1 to form a functional protein complex with MRAP2 but not MRAP1 in live cells, we performed bimolecular fluorescence complementation (BiFC) assays. To achieve this goal, we generated MCHR1 constructs fused to the YFP-F1 fragment in the C-terminus, while the C-terminal of MRAP1 or MRAP2 was fused to the complementary YFP-F2 fragment and Flag tag (**Figures 2E, F**). As expected, the expression of MRAP1-Flag-F2 with MCHR1-F1 alone did not exhibit any fluorescent signal. However, YFP fluorescence was detected when MCHR1-F1 and MRAP2-Flag-F2 were co-transfected, suggesting that MRAP2 but not MRAP1 could form a functional complex with MCHR1, because of the fact that the emission of a fluorescent YFP molecule required the close proximity of YFP-F1 and YFP-F2 fragments.

### Modulation of the Surface Translocation of MCHR1 by MRAP2 Proteins

**Figure 3:**
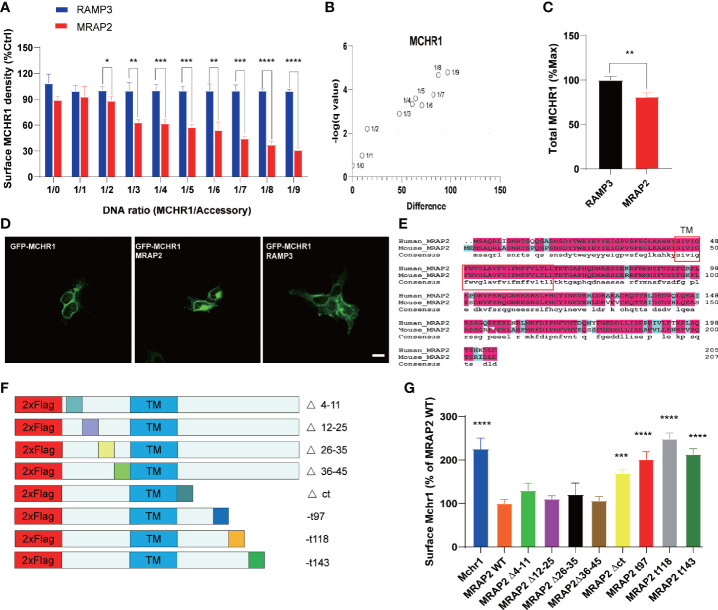
Regulation of MCHR1 trafficking by WT MRAP2 and its mutants. **(A)** Surface expression of MCHR1 measured by ELISA assay, which transfected with MRAP2 at different ratios (from 1:0 to 1:9). **(B)** D-value of MCHR1 membrane surface expression in the control group (RAMP3) and MRAP2 group at different transfection ratios. **(C)** Total expression level of MCHR1. **(D)** Localization of GFP-MCHR1 by confocal microscopy, which transfected with empty vector (left) or MRAP2 (middle) or RAMP3 (right). **(E)** Sequence alignment of human and mouse MRAP2. **(F)** Schematic representation of the MRAP2 mutants constructed. **(G)** The surface expression of MCHR1 when co-transfected with empty vector (control), WT MRAP2, or different mutants. One-way ANOVA with post-hoc Tukey test. *p < 0.05, **p < 0.01, ***p < 0.001, ****p < 0.0001.

The trafficking of several GPCRs has been shown to be modulated by MRAP2 (7, 12–14). To test whether MRAP2 alters the membrane translocation of MCHR1, we performed an enzyme-linked immunosorbent assay (ELISA) to measure the cell surface receptor’s expression quantitatively. The 3HA tag was added to the N-terminus of MCHR1 and the construct was then expressed with or without MRAP2 in HEK293T cells. To determine the effect of MRAP2 on the surface expression of MCHR1, 3HA-MCHR1 and either MRAP2 or RAMP3 (as a non-interacting negative control, another reported transmembrane protein that does not regulate GPCR signaling) at different receptor-to-accessory ratios (from 1:0 to 1:9) were transfected simultaneously. MCHR1 surface expression was detected at OD 450 nm upon the addition of the HA antibody and substrate with or without cell lysis blocking. Cell numbers were measured at OD 595 nm and normalized by Janus Green cell normalization stain. We showed that MRAP2 significantly decreased the surface expression of MCHR1 compared to the control group (**Figure 3A**). In general, as the ratio increased, the difference between the MRAP2 group and the control group increased (**Figure 3B**). These results indicated that more than two molecules of MRAP2 interacted with MCHR1, which was consistent with the results of OX1R studies. Our results showed that, at a 1:9 ratio, while MRAP2 decreased the maximal surface density of MCHR1 by >60%, the total expression of MCHR1 only reduced by 20% when MRAP2 existed compared to the control group (**Figure 3C**). GFP-MCHR1 alone, or with MRAP2 or RAMP3, was transfected to further visualize the impact of MRAP2 on MCHR1 localization. From the images with expression of GFP-MCHR1 only or with RAMP3, GFP fluorescence was mainly presented at the plasma membrane (**Figure 3D**, left and right panels), whereas GFP-MCHR1 was mostly retained in the intracellular compartment in the presence of MRAP2, which was consistent with the ELISA results (**Figure 3D**, middle panel). MCHR1 was reported to activate the ERK signaling pathway as a downstream signal (18, 19). To determine the effects of MRAP2 on downstream signaling cascades of MCHR1, we monitored ERK1/2 phosphorylation upon activation of MCHR1 by the treatment of MCH as seen in **Figure 2G**. As expected, we could see the significantly decreased levels of pERK1/2 in our dual-expressing cell lines, thus proving that MRAP2 downregulated Erk pathway upon activation of MCHR1.

### Repression of the Surface Expression of MCHR1 by Specific Regions of MRAP2

These preferential localizations suggested that MRAP2 was involved in MCHR1 trafficking to the plasma membrane. However, the functional domain of MRAP2 on regulating MCHR1 trafficking needed to be further explored. Several studies have shown that specific regions of MRAP2 are not required for the regulation of different GPCRs (9, 13). To localize the regions of MRAP2 that were essential for its inhibitory effect on MCHR1 trafficking, a series of MRAP2 mutants were artificially generated, in which the N-terminal fragments were deleted and the C-terminal fragments were truncated (**Figure 3F**). The truncated regions of mouse MRAP2 were the same as the human MRAP2 in a previous study (13), since we wanted to conduct an amino acid sequence comparison of mouse and human MRAP2. We also found that they were highly conservative, especially the TM domains (**Figure 3E**). By performing ELISA assay, we explored the effect of each MRAP2 mutant on the surface expression of MCHR1. Our results showed that the deletion of the whole C-tail caused a total loss of MRAP2 function on MCHR1 surface expression (**Figure 3G**).

**Figure 4:**
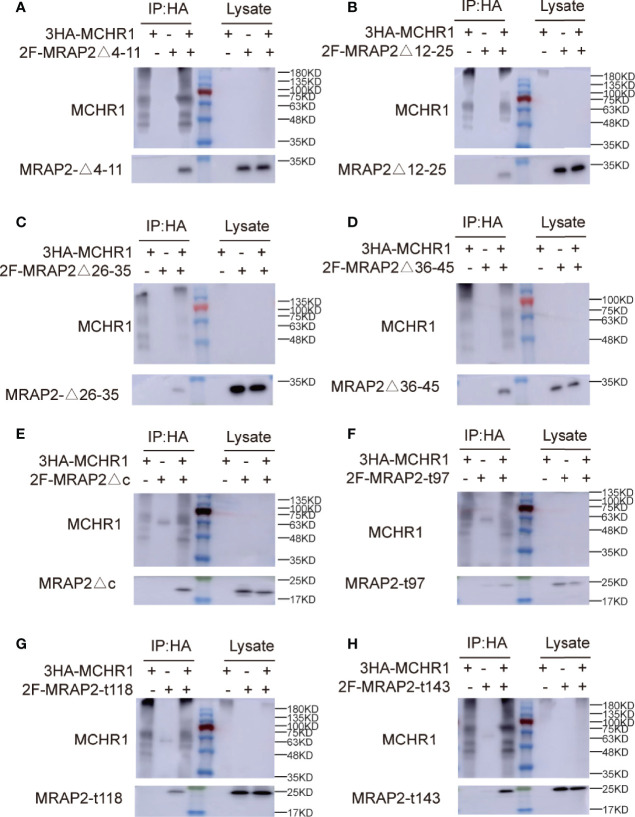
Interaction between 3HA-MCHR1 and MRAP2 mutants. **(A–D)** The interaction between MCHR1 and MRAP2 C-terminal mutants. **(E–H)** The interaction between MCHR1 and MRAP2 N-terminal mutants. Middle lane with color is protein marker in all figures. The numbers on the right indicate molecular weight of marker band in the middle (kd).

To determine whether the effect of these MRAP2 mutants on receptor membrane transport was caused by the disappearance of protein interaction, we performed Co-IP experiments to verify the interplay between each MRAP2 mutant with MCHR1. The results showed that each MRAP2 mutant still exhibited interactions with MCHR1 (**Figure 4**). Here, TM dimerization domain was still retained in all MRAP2 constructs (20) because Chen and his colleagues identified the TM as the essential domains for protein dimerization.

### Co-Localization of MRAP2 Mutants With MCHR1

**Figure 5:**
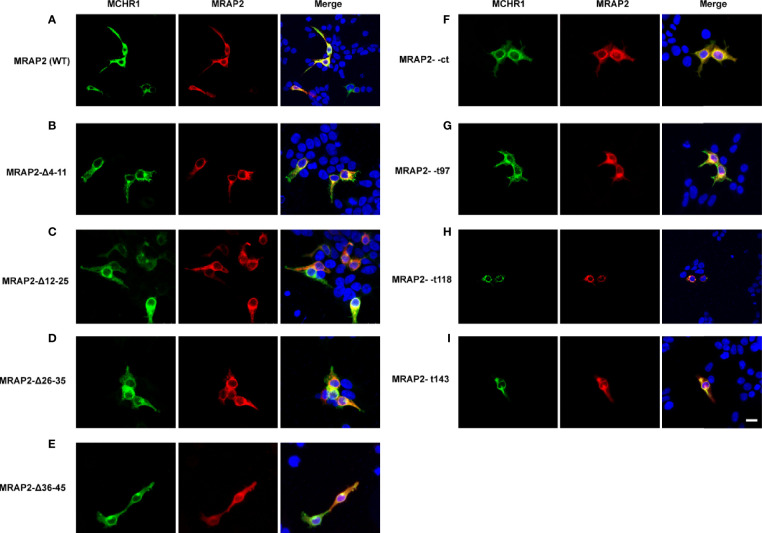
Co-localization of MRAP2 mutants with MCHR1. **(A)** Confocal images of MCHR1 (green) when co-transfected with WTMRAP2 (red). **(B–E)** Localization of MCHR1 (green) when co-transfected with MRAP2 C-terminal mutants (red). **(F–I)** Images are co-transfected with MCHR1 (green) and MRAP2 N-terminal mutants. DAPI was used to stain cell nuclei and shown in blue. Merged images are shown in yellow.

Next, we also performed co-immunofluorescence to investigate the functional regions of MRAP2 on modulating MCHR1 trafficking. Different MRAP2 mutants tagged with 2Flag (WT, Δ4–11, Δ12–25, Δ26–35, Δ36–45, Δct, t97, t118, and t143) were co-transfected with 3HA-MCHR1. The results showed that the nonfunctional MRAP2 mutants were all properly expressed (**Figure 5**), suggesting that the loss of pharmacological activity of these mutants was not due to the lack of expression. Moreover, the Co-IF results further confirmed that each MRAP2 mutant interacted actively with MCHR1.

### The Influence of MCHR1 Signaling by the Functional Domains of MRAP2

**Figure 6:**
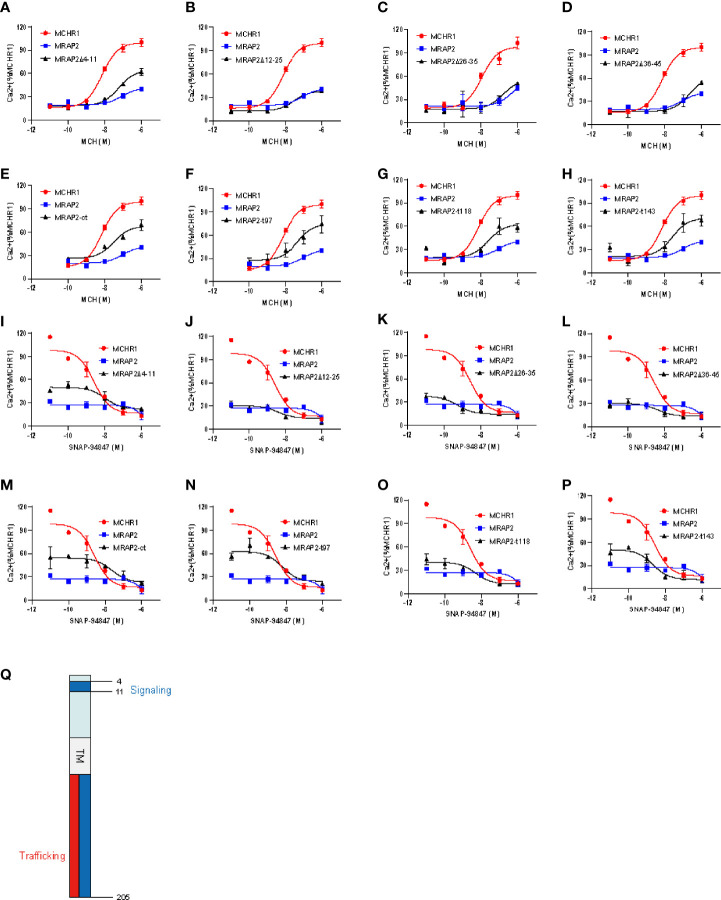
The influence of MCHR1 signaling by the functional domains of MRAP2. **(A–H)** Calcium response of MCHR1 simulated by increasing concentrations of MCH, transfected with WT MRAP2 or indicated mutants. **(I–P)** Competition binding assay of MCHR1 in 293T cells transfected with wt MRAP2 or indicated mutants. Relative luminescence intensity of NFAT-luc represents the normalized NFAT-luc units to p-RL-TK units (transfected internal control). Each data point represents the mean ± SEM of three replicates (*N* = 3). **(Q)** Schematic diagram of distinct regions of MRAP2 required for regulating the trafficking and signaling of MCHR1.

It was previously shown that MRAP2 inhibited the signaling of OX1R and PKR1 (13). Here, we tested whether these regions of MRAP2 were required for the inhibition of MCHR1. To achieve this goal, we transfected HEK293T cells with MCHR1 in the presence of WT MRAP2 or MRAP2 mutants at a 1:9 ratio and measured the Ca^2+^ influx of MCHR1 by CRE-luciferase reporter assay. The results showed that MRAP2 strongly decreased the agonistic efficacy of MCH (**Figures 6A–H**: the blue curve). Moreover, MRAP2-Δ12–45 retained a significant inhibitory action on MCHR1 signaling (**Figures 6B–D**: the black curve), while the inhibition of MRAP2 on MCHR1 signaling was almost completely reversed when transfected with MRAP2-Δ4–11 or the C-terminal truncations of MRAP2 (**Figures 6A, E–H**: the black curve). As shown in **Table 1**, the sensitivity of MCHR1 to MCH was reduced to varying degrees by addition of MRAP2 or MRAP2 truncated constructs. Especially with the addition of mMRAP2 Δ12-25 and Δ36-45 mutants, the EC50 value changed by an order of magnitude, from 10^-8^ M to 10^-7^ M.

**Table 1:** Statistical analysis of MCHR1 in the presence of different MRAP2 mutants in response to MCH.

Data statistics of **Figures 6A–H**	LogEC50	*p*-value comparison	
		*vs*. receptor alone	*vs*. MRAP2 WT
MCHR1 alone	−8.149 ± 0.10	–	<0.0001
MCHR1:MRAP2 WT	−7.061 ± 0.40	<0.0001	–
MCHR1:MRAP2 Δ4–11	−7.242 ± 0.19	<0.0001	<0.0001
MCHR1:MRAP2 Δ12–25	−6.765 ± 1.15	<0.0001	0.9803
MCHR1:MRAP2 Δ26–35	−7.551 ± 0.31	<0.0001	<0.0001
MCHR1:MRAP2 Δ36–45	−6.643 ± 0.43	<0.0001	0.8704
MCHR1:MRAP2 Δct	−7.515 ± 0.54	<0.0001	<0.0001
MCHR1:MRAP2 t97	−7.338 ± 0.68	<0.0001	<0.0001
MCHR1:MRAP2 t118	−7.593 ± 0.63	<0.0001	<0.0001
MCHR1:MRAP2 t143	−7.599 ± 0.46	<0.0001	<0.0001

Values were expressed as the mean ± S.E.M. of at least three independent experiments. Two-way ANOVA with Tukey post-test was applied in the statistical analysis.

In addition, we also performed competitive inhibition analysis (**Figures 6I–P**). We added different concentrations of MCHR1 antagonist (SNAP-94847) in the presence of EC80 MCH (10^-7^ M) to observe the effect of MRAP2 mutants on MCHR1. We found that consistent with the results observed in **Figures 6A–H**, the inhibition of MCHR1 signaling relied on the 4–11 amino acids and the C-terminus of MRAP2 (**Figures 6I, M–P**). Except for the addition of MRAP2 Δ 26-35 and T143, IC50 of MCHR1 slightly decreased, other variants all increased the sensitivity of MCHR1 to the inverse agonist, but in general, IC50 values were not significantly altered (**Table 2**). In short, these results indicated that the trafficking and signaling of MCHR1 were both inhibited by MRAP2, suggesting that different MRAP2 regions were involved in regulating distinct GPCRs (**Figure 6Q**).

**Table 2:** Statistical analysis of MCHR1 in the presence of different MRAP2 mutants in response to the antagonist of MCHR1.

Data statistics of **Figures 6I–P**	LogIC50	*p*-value comparison	
		*vs*. receptor alone	*vs*. MRAP2 WT
MCHR1 alone	−8.584 ± 0.53	–	<0.0001
MCHR1:MRAP2 WT	−7.752 ± 0.68	<0.0001	–
MCHR1:MRAP2 Δ4–11	−7.977 ± 0.57	<0.0001	<0.0001
MCHR1:MRAP2 Δ12–25	−8.451 ± 0.89	<0.0001	0.2763
MCHR1:MRAP2 Δ26–35	−9.278 ± 0.57	<0.0001	0.6027
MCHR1:MRAP2 Δ36–45	−8.408 ± 0.68	<0.0001	0.2858
MCHR1:MRAP2 Δct	−7.641 ± 0.94	0.0002	<0.0001
MCHR1:MRAP2 t97	−8.251 ± 0.69	0.0002	<0.0001
MCHR1:MRAP2 t118	−8.314 ± 0.69	<0.0001	0.4248
MCHR1:MRAP2 t143	−8.683 ± 0.51	<0.0001	0.0875

Values were expressed as the mean ± S.E.M. of at least three independent experiments. Two-way ANOVA with Tukey post-test was applied in the statistical analysis.

## Discussion

MRAP2 is an essential accessory factor for the regulation of melanocortin receptor family. Melanocortin receptor 4 (MC4R) is the first reported energy homeostasis-related GPCR that was regulated by MRAP2. MRAP2 potentiates MC4R signaling *in vitro* and the deletion of MC4R and MRAP2 both develop severe obesity *in vivo* (9, 21). MRAP2 was previously confirmed to be involved in the regulation of several other non-melanocortin receptors related to metabolism, such as preprokineticin receptor family (PKRs) (12), pro-orexin receptor 1 (OX1R) (13), and growth hormone secretagogue receptor 1a (GHSR1a) (9, 14). In this study, we further proved that MRAP2 functioned as a wide modulator of GPCRs and more receptors would be identified as MRAP2 partners in the near future.

MRAP2 owns a short N-terminus that contains a glycosylation site, and forms special antiparallel dimer structurally. The orthologs of MRAP1 have identical N-terminal and transmembrane (TM) regions, whereas the C-terminal portion is variable (22). Unlike MRAP1, the entire sequence of MRAP2 is highly conservative, including the C-termini, suggesting that the C-tail of MRAP2 performs some important functions (23). It has been proved that distinct regions of MRAP2 take part in the inhibition of trafficking and signal transduction of PKR1 and OX1R independently (13). Recently, the influences of MRAP2 on the Gα/Gq and β-arrestin pathways were also independent on relevant different regions of MRAP2 described by Rouault et al. (9). In our study, we identified the C-terminus as required for MRAP2 to repress the surface expression and inhibition of the activity of MCHR1, while for the N-terminal of MRAP2, only residues 4–11 exhibited important roles for modulating MCHR1 signaling. Taken together, the C-terminus seems to play a master role in regulating MCHR1. It is also noted that MRAP2-Δ4-11, which is functionally along with the C-terminal truncation of MRAP2 as those involved in inhibiting MCHR1 expression, may act *via* a different mechanism of action. As previously reported, a glycosylated asparagine at position 9, which was inactivated or deleted, could result into the functional loss of MRAP2. Also, we showed that the interaction between MCHR1 and MRAP2 still occurred upon deletion or mutation of the C-terminal and N-terminal, respectively. These results make sense, because it has been reported that the TM region of MRAP2 is the smallest region for MRAP2 to form homodimers (20), suggesting that the TM region is the key region for the interaction with GPCR and is relatively independent of the region for signal activity and membrane transport.

So far, all GPCRs regulated by MRAP2 and related to energy balance and food intake share a common feature, that is, highly expressed in the hypothalamus along with MRAP2. MRAP2 could regulate the sensitivity of a series of hypothalamic neurons to their neuropeptides, such as α-MSH, prokineticins, orexins, and Ghrelin. MCH is also abundant in the hypothalamus. Leptin appears to be an important biological signal in this MRAP2-GPCR system through which α-MSH/MCH additively or synergistically interacts to affect food intake and body weight regulation. When leptin binds to its receptors on POMC neurons, POMC releases α-MSH (α-melanocortin stimulating hormone), which activates downstream MC4R to transmit satiety signals (24), and MRAP2 interacts with MC4R and potentiates its activity, thereby inhibiting food intake. Leptin reduced the expression of MCHR1 and ob/ob mice significantly increased MCHR1 expression in the hypothalamus (25). MCH and α-MSH show the opposing effects in which MCHR1 expression is decreased because MCH is repressed by leptin. Functionally, although MRAP2 inhibits surface expression of MCHR1, it is unclear how this could affect physiological processes of food intake. We speculate that MRAP2 inhibits MCHR1 signaling, and might lead to food intake reduction, which needs to be further confirmed *in vivo*. Based on some recent work demonstrating that MCHR1 is abundantly expressed in nerve cilia and in which it regulates cilia length and signaling pathways (26, 27), we are also interested in whether MRAP2 regulates MCHR1 function in nerve cilia *in vivo* in the future. In short, our work reveals mechanisms of the MRAP2 functional domains involved in GPCR surface expression and activity. The identification of MRAP2 interacting partners shows novel aspects of physiological control and may benefit the pharmaceutical pipelines to develop drugs to target these pathways in future studies.

## Data Availability Statement

The datasets presented in this study can be found in online repositories. The names of the repository/repositories and accession number(s) can be found in the article/supplementary material.

## Ethics Statement

The animal study was reviewed and approved by the Department of Plastic and Reconstructive Surgery, Shanghai Institute of Precision Medicine, Shanghai Ninth People’s Hospital, Shanghai Jiao Tong University School of Medicine, Shanghai, China.

## Author Contributions

ChZ conceived and designed the study. MW, JX, XL, and BJ performed the experiments. YZ, CoZ, SL, and ZK performed data analysis. TZ, L-NJ, QL, and ChZ are responsible for writing and revising manuscript. All authors contributed to the article and approved the submitted version.

## Funding

This work was supported by grants from the National Key Research and Development Program of China (Grant Nos. 2017YFA0103902 and 2019YFA0111400), Shanghai Municipal Key Clinical Specialty (Grant No. shslczdzk00901), the Innovative Research Team of High-level Local Universities in Shanghai (Grant No. SSMU-ZDCX20180700), and the Key Laboratory Program of the Education Commission of Shanghai Municipality (ZDSYS14005).

## Conflict of Interest

The authors declare that the research was conducted in the absence of any commercial or financial relationships that could be construed as a potential conflict of interest.

## Publisher’s Note

All claims expressed in this article are solely those of the authors and do not necessarily represent those of their affiliated organizations, or those of the publisher, the editors and the reviewers. Any product that may be evaluated in this article, or claim that may be made by its manufacturer, is not guaranteed or endorsed by the publisher.

## References

[1] Bohlooly YM Mahlapuu M Andersen H Astrand A Hjorth S Svensson L. Osteoporosis in MCHR1-Deficient Mice. Biochem Biophys Res Commun (2004) 318:964–9. doi: 10.1016/j.bbrc.2004.04.122 15147966

[2] Zhang LN Sinclair R Selman C Mitchell S Morgan D Clapham JC. Effects of a Specific MCHR1 Antagonist (GW803430) on Energy Budget and Glucose Metabolism in Diet-Induced Obese Mice. Obes (Silver Spring) (2014) 22:681–90. doi: 10.1002/oby.20418 23512845

[3] Antal-Zimanyi I Khawaja X. The Role of Melanin-Concentrating Hormone in Energy Homeostasis and Mood Disorders. J Mol Neurosci (2009) 39:86–98. doi: 10.1007/s12031-009-9207-6 19418262

[4] Kowalski TJ Spar BD Weig B Farley C Cook J Ghibaudi L. Effects of a Selective Melanin-Concentrating Hormone 1 Receptor Antagonist on Food Intake and Energy Homeostasis in Diet-Induced Obese Mice. Eur J Pharmacol (2006) 535:182–91. doi: 10.1016/j.ejphar.2006.01.062 16540104

[5] Elliott JC Harrold JA Brodin P Enquist K Backman A Bystrom M. Increases in Melanin-Concentrating Hormone and MCH Receptor Levels in the Hypothalamus of Dietary-Obese Rats. Brain Res Mol Brain Res (2004) 128:150–9. doi: 10.1016/j.molbrainres.2004.06.010 15363890

[6] Asai M Ramachandrappa S Joachim M Shen Y Zhang R Nuthalapati N. Loss of Function of the Melanocortin 2 Receptor Accessory Protein 2 Is Associated With Mammalian Obesity. Science (New York NY) (2013) 341:275–8. doi: 10.1126/science.1233000 PMC3788688 23869016

[7] Chan LF Webb TR Chung TT Meimaridou E Cooray SN Guasti L. MRAP and MRAP2 Are Bidirectional Regulators of the Melanocortin Receptor Family. Proc Natl Acad Sci USA (2009) 106:6146–51. doi: 10.1073/pnas.0809918106 PMC2661846 19329486

[8] Huszar D Lynch CA Fairchild-Huntress V Dunmore JH Fang Q Berkemeier LR. Targeted Disruption of the Melanocortin-4 Receptor Results in Obesity in Mice. Cell (1997) 88:131–41. doi: 10.1016/S0092-8674(00)81865-6 9019399

[9] Sebag JA Zhang C Hinkle PM Bradshaw AM Cone RD. Developmental Control of the Melanocortin-4 Receptor by MRAP2 Proteins in Zebrafish. Science (New York NY) (2013) 341:278–81. doi: 10.1126/science.1232995 PMC4255277 23869017

[10] Baron M Maillet J Huyvaert M Dechaume A Boutry R Loiselle H. Loss-Of-Function Mutations in MRAP2 Are Pathogenic in Hyperphagic Obesity With Hyperglycemia and Hypertension. Nat Med (2019) 25:1733–8. doi: 10.1038/s41591-019-0622-0 PMC6858878 31700171

[11] Hinney A Schmidt A Nottebom K Heibült O Becker I Ziegler A. Several Mutations in the Melanocortin-4 Receptor Gene Including a Nonsense and a Frameshift Mutation Associated With Dominantly Inherited Obesity in Humans. J Clin Endocrinol Metab (1999) 84:1483–6. doi: 10.1210/jcem.84.4.5728 10199800

[12] Chaly AL Srisai D Gardner EE Sebag JA. The Melanocortin Receptor Accessory Protein 2 Promotes Food Intake Through Inhibition of the Prokineticin Receptor-1. eLife (2016) 5:e12397. doi: 10.7554/eLife.12397 26829592 PMC4786424

[13] Rouault AAJ Lee AA Sebag JA. Regions of MRAP2 Required for the Inhibition of Orexin and Prokineticin Receptor Signaling. Biochim Biophys Acta (2017) 1864:2322–9. doi: 10.1016/j.bbamcr.2017.09.008 28939058

[14] Srisai D Yin TC Lee AA Rouault AAJ Pearson NA Grobe JL. MRAP2 Regulates Ghrelin Receptor Signaling and Hunger Sensing. Nat Commun (2017) 8:713. doi: 10.1038/s41467-017-00747-6 28959025 PMC5620068

[15] Rouault AAJ Rosselli-Murai LK Hernandez CC Gimenez LE Tall GG Sebag JA. The GPCR Accessory Protein MRAP2 Regulates Both Biased Signaling and Constitutive Activity of the Ghrelin Receptor GHSR1a. Sci Signaling (2020) 13(613):eaax4569. doi: 10.1126/scisignal.aax4569 PMC7291826 31911434

[16] Agulleiro MJ Roy S Sanchez E Puchol S Gallo-Payet N Cerda-Reverter JM. Role of Melanocortin Receptor Accessory Proteins in the Function of Zebrafish Melanocortin Receptor Type 2. Mol Cell Endocrinol (2010) 320:145–52. doi: 10.1016/j.mce.2010.01.032 20138960

[17] Jones BW Song GJ Greuber EK Hinkle PM. Phosphorylation of the Endogenous Thyrotropin-Releasing Hormone Receptor in Pituitary GH3 Cells and Pituitary Tissue Revealed by Phosphosite-Specific Antibodies. J Biol Chem (2007) 282:12893–906. doi: 10.1074/jbc.M610854200 17329249

[18] Eberle AN Mild G Schlumberger S Drozdz R Hintermann E Zumsteg U. Expression and Characterization of Melanin-Concentrating Hormone Receptors on Mammalian Cell Lines. Peptides (2004) 25:1585–95. doi: 10.1016/j.peptides.2004.06.022 15476925

[19] Pissios P Trombly DJ Tzameli I Maratos-Flier E. Melanin-Concentrating Hormone Receptor 1 Activates Extracellular Signal-Regulated Kinase and Synergizes With G(s)-Coupled Pathways. Endocrinology (2003) 144:3514–23. doi: 10.1210/en.2002-0004 12865333

[20] Chen V Bruno AE Britt LL Hernandez CC Gimenez LE Peisley A. Membrane Orientation and Oligomerization of the Melanocortin Receptor Accessory Protein 2. J Biol Chem (2020) 295:16370–9. doi: 10.1074/jbc.RA120.015482 PMC7705299 32943551

[21] Asai M. Loss of Function of the Melanocortin 2 Receptor Accessory Protein 2 Is Associated With Mammalian Obesity (Vol 341, Pg 275, 2013). Science (New York NY) (2013) 341:959–9. doi: 10.1126/science.1233000 PMC3788688 23869016

[22] Webb TR Clark AJ. Minireview: The Melanocortin 2 Receptor Accessory Proteins. Mol Endocrinol (2010) 24:475–84. doi: 10.1210/me.2009-0283 PMC5419097 19855089

[23] Rouault AAJ Srinivasan DK Yin TC Lee AA Sebag JA. Melanocortin Receptor Accessory Proteins (MRAPs): Functions in the Melanocortin System and Beyond. Biochim Biophys Acta (2017) 1863:2462–7. doi: 10.1016/j.bbadis.2017.05.008 28499989

[24] Ranadive SA Vaisse C. Lessons From Extreme Human Obesity: Monogenic Disorders. Endocrinol Metab Clin North Am (2008) 37:733–51. doi: 10.1016/j.ecl.2008.07.003 PMC5877402 18775361

[25] Kokkotou EG Tritos NA Mastaitis JW Slieker L Maratos-Flier E. Melanin-Concentrating Hormone Receptor Is a Target of Leptin Action in the Mouse Brain. Endocrinology (2001) 142:680–6. doi: 10.1210/endo.142.2.7981 11159839

[26] Alhassen W Kobayashi Y Su J Robbins B Nguyen H Myint T. Regulation of Brain Primary Cilia Length by MCH Signaling: Evidence From Pharmacological, Genetic, Optogenetic, and Chemogenic Manipulations. Mol Neurobiol (2022) 59(1):245–65. doi: 10.21203/rs.3.rs-485543/v1 PMC9083846 34665407

[27] Hsiao YC Muñoz-Estrada J Tuz K Ferland RJ. The Transition Zone Protein AHI1 Regulates Neuronal Ciliary Trafficking of MCHR1 and Its Downstream Signaling Pathway. J Neurosci Off J Soc Neurosci (2021) 41:3932–43. doi: 10.1523/JNEUROSCI.2993-20.2021 PMC8084322 33741721

